# Supplementary material for: Are carbon emissions trading and green financial instruments synergistic? -Comprehensive quantitative research based on content analysis
Source: PLoS One. 2024 Mar 7;19(3):e0298601. doi: 10.1371/journal.pone.0298601 (PMC10919601; doi:10.1371/journal.pone.0298601)
Supplement: S1 Data — (ZIP) [file pone.0298601.s001.zip › Data/地级市Z_F工作报告.zip!/╣π╢1⁄2/╖≡╔╜╩╨/└┤╫╘╣1⁄2╓┌║┼í╛┬φ┐╦╩2╛▌═°í┐_2013.docx]

各位代表：

　　现在，我代表佛山市人民政府向大会报告政府工作，请予审议，并请政协各位委员和其他列席人员提出意见。

　　一、2012年工作回顾

　　2012年，是本届政府任期的第一年，也是佛山经济面临复杂形势、发展遇到较大困难的一年。市政府在省委、省政府和市委的正确领导下，在市人大和市政协的监督支持下，坚持以科学发展观为指导，提振信心、团结奋进，扎实推动经济社会发展，各项工作取得新成绩。全市实现生产总值6709.02亿元，增长8.2%；财政总收入1261.7亿元，增长13.55%，其中地方公共财政预算收入384.08亿元，增长12.39%；城镇居民人均可支配收入34580元，农村居民人均纯收入15684元，分别增长12.6%和13.1%；居民消费价格涨幅2.6%；人口自然增长率5.78％。。

　　一年来，我们主要做了以下工作：

　　（一）积极应对经济下行压力，经济保持平稳发展

　　开展“暖春活动”帮助企业走出困境。初步统计，全年为企业减免税费超过35亿元。争取中央、省专项资金18.15亿元，累计向中小企业融资452亿元、投放贷款161亿元。组织企业开拓内、外需市场。实施民营企业家素质提升工程，培训企业经营管理者400多人。

　　产业链招商成效明显。实施产业链招商三年行动计划，成功在上海、深圳举办产业链对接合作洽谈会，共达成签约项目34个。在东盟、欧洲、台湾等国家和地区开展招商推介活动，全年实际利用外资23.5亿美元，同比增长9.07%；签约超千万美元外资项目56个，引进世界500强项目14个。国药集团中药产业基地、台湾旺旺集团华南生产基地、亨氏集团、安德里茨设备制造中心、广东富华工程机械制造等一批优质项目落户。

　　产业结构调整取得实效。传统产业调整升级力度加大。实施“质量效益双提升”计划，机械装备、陶瓷等四大行业70个技改重点项目推进顺利。推动“两化”深度融合，46个项目被认定为省级示范项目，顺德区成功申报国家级装备工业两化深度融合暨智能制造试点。扎实推进“双转移”，产业转移工作在全省考核中获优秀等次。先进制造业和新兴产业发展态势良好。一汽大众主机厂基本建成，广东新光源产业基地新增企业36家。现代服务业发展有新突破。引进华强电子信息高端服务业基地、绿地集团城市综合体等一批重大项目，海尔（三水）产业园物流中心投入运营，广东物联天下物联网信息产业园顺利开园。国家旅游产业集聚实验区落户南海，广东现代服务业集聚区在三水新城正式挂牌，西樵山、皂幕山分别创建为国家5A、4A级景区。现代农业加快发展，现代农业园区增至28个，省级以上农业龙头企业达14家。

　　高端产业载体建设实现新突破。中德工业服务区被列入省六大重点发展平台之一，并上升到中德两国国家级合作层面。佛山高新区实施“一区五园”新体制，核心园区落户南海狮山和三水乐平。广东金融高新区致力发展产业金融，引进各类机构和项目 104个。中国南方智谷被认定为国家现代服务业科技服务产业化基地，入驻创新团队13个。广东工业设计城入选粤港澳科技创新合作示范区服务贸易自由化重点项目。智慧新城核心区完成总投资的91%，格力电器、日立电梯等一批大型企业落户。

　　（二）推动科技金融产业融合发展，产业核心竞争力得到增强

　　科技引领发展作用日益凸显。创建国家创新型城市工作全面启动。以企业为主体的科技创新活动持续开展，全年组织企业申报各类科技项目1300多项，全市新增国家级企业技术中心2家、省级工程技术研究开发中心5家，新建产业技术创新联盟6个。产学研合作成效显著，共获得省部产学研结合项目52项、省院战略合作项目28项、院市合作项目70项。与中科院合作3年多来，引入创新团队41个、育成企业41家，佛山中科院产业技术研究院进驻佛山新城。与德国弗劳恩霍夫协会签署框架协议，共建中德工业服务区。品牌、标准、专利战略有效推进，新增各类标准92项；专利授权17839件，增长9.1%，其中发明专利授权1161件，增长19.4%。

　　金融创新发展不断深化。出台促进金融服务实体经济的若干意见，提出试点设立科技金融机构、推广“商圈”融资模式等创新举措服务实体经济。全市新增上市公司5家，总数达37家；新增股权投资基金65支，总数达125支。外资银行在佛山设立分支机构11家。探索民间金融创新发展新路径，市民营企业投资商会成立，佛商壹号投资基金启动。南海区成为全国首个通过知识产权质押融资验收的试点城市，佛山成为全省首个实现行政村金融服务全覆盖的地级市。

　　（三）全力推进城市建设和管理，城市品质稳步提升

　　城市升级引领城市蜕变。国务院批准我市土地利用总体规划,明确中心城区规划控制土地总面积为361.66平方公里。实施城市升级三年行动计划，开工67个项目，建成15个项目。落实“强中心”战略，“一老三新”[1]中心城区及其他组团城区升级步伐加快。禅城老城区改造提升成效明显。岭南天地二期基本建成，中山公园升级工程启动，升平路、永安路、南堤路商业街主体工程完工。佛山新城建设加速提质。佛山新地标苏宁广场等中央商务区八大项目同步奠基，滨江景观带初步建成。桂城千灯湖片区建设进展顺利。海八路金融隧道建成通车，平洲玉器城特色步行街区一期完工。禅西新区建设稳步推进。南庄岭南生态园中心湖河网工程全面完工，东片区主要道路工程基本建成。顺德新城43个重点项目全面铺开。西江新城完成5亿元建设量，君御温德姆至尊酒店如期封顶。三水新城规划编制基本完成。城市轴线和节点景观改造亮点纷呈。创建“全国绿化模范城市”通过国家核查组认定达标，生态景观林带建设取得阶段性成果。禅城区加大城市道路“五位一体”改造[2]力度，祖庙路、锦华路等道路改造完工，佛山大道改造工程进展顺利，佛山火车站区域提升成效明显，广佛轨道出入口绿化景观提升全面完成。南海区大沥镇广佛路改造、广佛新干线（一期）绿化工程、佛山水道沥桂新城区段滨河景观提升等顺利完成，里水河一河三岸景观塑造日见成效，华南电光源灯饰城外立面改造成为样板。顺德区对外主要道路衔接口、广珠西线及顺德站绿化景观提升完工，乐从新桂路及杏坛城市道路改造成效显现。高明区丽江水廊、秀丽河堤围景观综合整治初见成效，沧江路改造成为示范。三水区广三高速城区段两旁美化亮化、西南城区重点地段亮化等工程完成，一环东路美化绿化成为样板工程。交通基础设施建设加强。广三、佛开高速扩建任务完成，碧桂路改造、魁奇路东延线一期主线、一环南延线等建成通车，汾江路及岭南大道南延线、广佛轨道二期动工。大气和水环境治理力度加大，小燃煤锅炉、VOC[3]、黄标车、黑烟车进一步整治，节能降耗成效明显。佛山获全国城市供电可靠性[4]A级金牌，三水区成功申报国家新能源示范城市，禅城区低碳发展经验入选第18届全球气候大会。“三旧”改造深入推进。广佛国际商贸城、国际家居博览城、高明三洲旧区等项目改造成效显著。

　　城市管理开创新局面。出台加强城市管理的政策文件，设立市城管委，开展以市政环卫、户外广告、建筑工地、农贸市场等为重点的专项整治，城市重点区域和路段“脏乱差”现象明显改观。禅城区清理卫生死角800多个，华远东路乱堆放、惠景路占道经营等得到治理，佛山火车站、粤运汽车站、城北批发市场等周边市容明显好转。南海区开展违章广告牌和建筑工地整治，广佛高速两侧违章广告牌已清拆，建筑工地乱洒漏现象得到治理。顺德区开展“美城行动”，建筑工地周边环境得到整治。高明区沧江路等路段户外广告、高明影院门前乱搭建治理取得成效。三水区西南市场、德保市场等乱摆卖得到整治，中心城区车辆乱停放问题有效缓解。佛山新城河堤路乱洒漏现象得到控制，新乐路车辆乱停放得到治理。启动137个农贸市场升级改造，完工109个。采取“管建引限”[5]措施，推动中心城区治堵取得初步成效。公共交通加快发展，全市城区公交分担率提高到20%。智慧城市建设不断加强，市民融合服务平台正式启动，市民办事可享受一站式便捷服务。

　　城市文化品质稳步提升。佛山新城图书馆、档案中心、科技馆、青少年宫以及艺术村基本建成。全国文化信息资源共享工程基层服务点覆盖全市所有镇（街道），农家书屋实现行政村全覆盖，城乡四级公共文化服务网络日益完善。佛山粤剧《小凤仙》进入国家舞台艺术精品30强并晋京展演，秋色欢乐节、“寻梦佛山”异地务工人员夏令营等文化活动精彩纷呈。大力扶持文化产业发展，西樵山梦工场一期、岭南文化苑等项目投入使用。

　　“创文”营造文明风尚。针对“两个测评体系”中存在的薄弱环节，积极开展文明创建活动，尤其是通过开展具有佛山特色的“微文明”系列品牌活动，倡导市民从细节、小事做起，实现城市“大文明”，国检中“创文”取得明显成果。

　　《规划纲要》四年大发展任务基本完成。“四年大发展”涉及我市的重要指标、重大项目年度任务基本完成。广佛同城化建设取得新进展，两市完成重点交界地区规划整合，黄榄干线接番顺公路、五沙大桥扩建有序推进，19家商业银行实现广佛间行内通存通兑免手续费。广佛肇经济圈建设取得突破，经济合作区总体规划通过专家评审，通信资费实现一体化，广佛肇城际轨道、三花公路改造一期等项目推进顺利。佛港澳合作进一步深化。

　　（四）着力加强社会民生事业，民生福祉持续改善

　　省市民生实事圆满完成。2012年全市财政投入民生方面资金262.58亿元，占地方公共财政预算支出的60.65%。两次提高企业退休人员养老金标准，月人均养老金从1570元提高到1940元。城乡最低生活保障标准全市统一提高为430元/人·月。新建平价商店115家。保障性住房开工7694套，竣工3920套，完成华侨农场职工危房改造200户。省级饮用水源保护标准化建设基本完成。中心城区垃圾分类试点初见成效，城镇生活垃圾无害化处理率达92%。建设64家“健康e园”。免费为市民发放佛山健康卡166.5万张。率先实现残疾儿童少年15年免费教育，启聪学校建设基本完成。免费孕前优生健康检查目标人群覆盖率达80%以上。首次将职工生育津贴纳入保障范围。实施流动人口计划生育节育奖励。基本实现户外体育健身设施行政村（社区）全覆盖。佛山新城露天泳场免费向市民开放。

　　社会事业全面进步。实施积极就业政策，全市新增城镇就业人数9.1万人，城镇登记失业率2.42%。合并实施新农保与城镇居民社会养老保险制度，提前实现两项社保全覆盖。启动社会养老服务体系建设年活动，居家养老服务工作力度加大。教育综合改革试点取得阶段性成果。学前教育三年计划积极实施，非户籍人口子女读书问题得到妥善解决，“数码学习港”全面启动，顺利通过广东省“推进教育现代化先进市”督导验收。公立医院综合改革试点稳步推进，基层医疗卫生服务网络进一步完善，“三平价”[6]医疗服务探索创新，全科医生制度顺利实施，“看病难、看病贵”问题得到缓解。成功举办国际龙舟邀请赛，亚洲龙舟联合会总部落户佛山。成功举办佛山首届公益慈善盛典暨“温爱佛山”慈善万人行活动。实施妇女儿童发展规划，稳步推进民族宗教工作，启动仁寿寺提升前期工作。援藏援疆、对口帮扶和革命老区“双到”工作任务成绩突出。

　　“三打两建”[7]成效显著。针对群众反映最强烈、问题最突出、社会危害最大的黑恶势力、保护伞和食品、药品等重点问题，采取有效措施，落实领导包案，狠打利益链，深挖保护伞，“三打”行动不断向纵深开展。坚持边打边建，启动“信用佛山”建设，强化市场监管，顺德区、南海区“两建”综合试点推进顺利。成功举办首届广东（佛山）安全食用农产品博览会，展示了“三打两建”中我省食品、农产品质量安全监管取得的成效。

　　社会大局稳定和谐。实施专业化精确打击战略，深入开展严打整治行动,加强社会治安综合治理，群众安全感稳步提高。安全生产“一岗双责”和“双基工作”走在全省前列，消防“网格化”管理模式全面推行，信访和公共安全等应急机制进一步健全，全年无重大安全事故发生。组织民兵预备役人员参与抢险救灾等活动，军政军民团结局面进一步巩固。

　　（五）不断深化各项改革，发展环境日益优化

　　行政管理体制改革走在全省前列。在全省率先启动行政管理体制改革试点，系统推进政府职能清理、商事登记制度改革、审批模式创新和审批流程再造等工作。市级部门通过取消、转移、下放、购买服务和优化调整等方式，共压减审批和管理事项536项，精简率达48.3%。全市逾550家企业完成商事注册登记。广东省网上办事大厅佛山分厅正式运营，全市网上办理业务量达30%以上，其中南海区达50%以上。跨部门并联审批使企业准入、工程验收审批时间均比改革前压缩50%以上，行政效能显著提升。

　　社会管理改革取得进展。完善社会建设相关政策体系。通过降低社会组织准入门槛、推行直接登记、建设社会组织孵化基地，促进社会组织蓬勃发展，全市共登记备案社会组织3549个。政府购买服务制度进一步健全，社工人才队伍建设得到加强。禅城区加快教育、医疗等重点领域公共事业体制改革，构建新型公共事业发展格局。顺德区社会创新园正式开园，全部镇（街道）建立了枢纽型社会服务综合体。

　　农村体制改革开创基层治理新格局。顺德区推动政府公共服务延伸村（居），启动法定机构试点改革，建立健全决策咨询机构，推进政社协同共治。南海区加快“政经分离”[8]农村综合改革步伐，明确基层组织权责边界，创新集体资产监管方式，引导村居回归自治。全市农村“两个平台”[9]建设圆满完成，农村“三资”管理[10]走向规范化。

　　财政国资改革取得实效。完善财政预算管理和绩效管理制度，强化预算执行刚性，扩大专项经费竞争性分配范围，财政管理科学化、精细化水平进一步提高。落实“大国资、全覆盖”总体工作方案，93家市属国有企业实现资产整合重组。建立市级土地储备财力保障机制，优化国有企业债务，国有企业可持续发展能力得到增强。

　　（六）大力加强政府自身建设，公务员素质和能力明显提高

　　法治政府建设得到加强。坚持向人大报告工作和向政协通报工作制度，自觉接受人大和政协的监督。认真办理人大代表议案、建议和政协提案。全面开展普法宣传和规范性文件清理，依法行政水平得到提高。开通佛山微博发布厅平台，政务信息公开更加透明。信访服务实现一站式接待、一条龙办理、一揽子解决。

　　公务员选用和考核机制进一步完善。面向全市公选处级领导干部，从优秀异地务工人员中选拔基层公务员，市直与区、镇（街道）公务员实行双向交流挂职。开展绩效管理试点，打造阳光政府和效能政府。

　　政府廉政建设成绩明显。成立市预防腐败局，强化廉政风险防控。建立重大决策部署效能监察联动机制、重点项目专责及责任倒逼机制，公共资源交易平台建设和监管进一步加强。开展公共服务行业民主评议政风行风工作，政风行风进一步好转。加强对重点企事业单位巡查，监管制度进一步完善。加大查办案件工作力度，坚决查处了一批违纪违法案件。

　　与此同时，审计、统计、粮食储备、外事侨务、台湾事务、档案、方志、人防、气象等工作都取得了新成绩。

　　各位代表！过去一年，我们与全市人民风雨同舟、携手共进，成绩来之不易。在此，我代表市政府向全市广大干部群众，外来务工人员，中央、省驻佛山单位，驻佛山人民解放军指战员、武警官兵表示崇高的敬意！向长期关心支持佛山建设的港澳台同胞、海外侨胞、国际友人表示衷心的感谢！

　　总结过去的工作，我们也清醒地看到，我市经济社会发展仍然存在一些问题，主要表现在：一是受外部环境和自身经济结构影响，去年地区生产总值增速未达到10%预期目标；二是产业结构调整任务艰巨，传统产业比重依然过大，战略性新兴产业尚未成长为经济发展重要支撑；三是创新能力不强，科技研发投入力度不足，人才短板成为发展制肘；四是“城市病”仍待破解，交通拥堵、环境污染、土地资源紧缺、局部电网薄弱等问题依然突出，节能减排、治水治气任重道远；五是和谐社会仍然存在局部隐患，社会建设有待加强；六是政府执行力和公信力有待进一步提高。对于存在的问题，我们必须在今后工作中切实加以解决。

　　二、2013年面临的形势和目标任务

　　2013年是全面贯彻落实党的十八大精神的开局之年，也是本届政府科学施政、创新发展实现新跨越的重要一年。

　　今年发展形势仍然复杂，面临的不利因素和挑战较多。但我们也应该看到，今年有利于发展的因素也在增多。纵观国际国内大势，我国发展仍处于可以大有作为的重要战略机遇期，新型工业化、城镇化、信息化和农业现代化为经济社会发展提供了长期充足的动力。党的十八大胜利召开，进一步激发了全国各地加快发展、科学发展的热情，为我们抢抓机遇、克难前进注入了强大信心。近年来我市围绕转变经济发展方式，推出了一系列创新性举措：一是引领改革潮流，深入推进行政、经济、社会等全方位改革，充分释放了体制机制活力，将逐步收获改革红利；二是多年来积累了雄厚的经济基础和众多产业投资项目，将逐步加大投资力度和释放产能，进一步增强实体经济竞争力；三是我市推出城市升级三年行动计划，实施“强中心”战略，推进智慧佛山建设，以工业化、城镇化、信息化、农业现代化共同驱动经济发展；四是我市提出建设国家创新型城市，以创新驱动支撑和引领佛山产业和城市升级，将推动佛山在科学发展轨道上阔步前行。对此，我们应对未来发展充满信心，以高度的责任感和只争朝夕的紧迫感推进各项工作，确保经济和社会平稳健康发展。

　　2013年政府工作的总体要求是：以党的十八大精神为指导，认真贯彻落实习近平总书记视察广东提出“三个定位、两个率先”指示和省委十一届二次全会、市委十一届四次全会精神，以提高经济增长质量和效益为中心，深化改革开放，坚持创新驱动，着力推进国家创新型城市建设，着力推进经济结构战略性调整，着力推进城市建设和管理，着力推进文化强市建设，着力推进生态文明建设，着力保障和改善民生，努力实现经济持续健康发展和社会和谐稳定，不断开创科学发展新局面。

　　2013年经济社会发展主要预期目标是：全市生产总值增长9%；地方公共财政预算收入增长10%；城镇居民人均可支配收入增长10%；农村居民人均纯收入增长11%；居民消费价格总水平涨幅控制在3.5%左右；城镇登记失业率控制在3.5%以内；人口自然增长率控制在7.64％。以内。

　　实现上述目标任务，我们要重点抓好以下八方面工作：

　　（一）着力推动国家创新型城市建设，增强可持续发展竞争力

　　科技创新是增强佛山核心竞争力的战略支撑。我们要大力实施创新驱动发展战略，把全市的智慧和力量凝聚到建设国家创新型城市上来。

　　明确创新型城市发展定位。通过创建，把佛山建设成为：国际高端制造业基地，国家产业技术创新高地，全国科技、金融、产业“三融合”示范区，国家创新驱动发展示范城市。

　　实施八项行动计划。一是投入百亿创新资金。从今年起，五年市区财政投入超过100亿元，带动企业和社会投入超过1000亿元。二是构建一流创新体系。包括一流的技术创新、现代产业、创新服务、创新人才、环境支撑体系。三是实施十大创新工程。包括企业创新主体提升、传统制造业创新升级、新兴产业培育提升、创新人才建设、科技金融产业融合、知识产权战略推进工程等。四是建设十大创新园区。高标准建设好创新创业的优质载体，打造佛山高新区、广东金融高新区、佛山国家火炬创新创业园、中国南方智谷、国家环境服务业华南集聚区、华南电源创新科技园等。五是打造十大创新平台。包括佛山中科院产业技术研究院和育成中心、广东西安交通大学研究院、顺德中山大学—卡内基梅隆大学国际联合研究院、广东半导体照明产业技术联合创新中心、华南精密研究院等。六是建设十大创新联盟。精选战略性新兴产业和优势传统产业，联合产业链上下游的企业与高校、科研院所共同组建十大产业技术创新战略联盟。七是开展一批项目全球招标。在优势传统产业和战略性新兴产业领域，遴选十大领域开展全球项目招标，重点突破一批产业发展重大关键共性技术，提升行业技术创新能力和产业竞争力。八是培育百强创新企业。从今年起，每年不少于20家，五年内精心培育100家综合实力较强的本土创新型企业。

　　营造良好创新创业环境。树立创新驱动核心理念，弘扬“和谐、开放、包容”的城市人文精神和“敢为人先、宽容失败”的创新创业文化。实施专利、科技创新团队、重大科技项目管理、科技创新平台资助等配套政策，加快形成完善的自主创新政策体系。强化创新成果保护，重拳打击知识产权侵权行为，提高知识产权保护和市场监管水平。

　　（二）着力推动产业转型升级，增强实体经济核心竞争力

　　实体经济是佛山经济发展的命脉。我们要继续加强产业转型升级，努力把佛山经济核心竞争力提高到新水平。

　　全力推动传统产业升级。通过行业准入标准或调整提升标准、节能减排、环境保护等倒逼机制，促进传统产业进行技术创新，推动产业从价值链低端向高端延伸。继续推动“两化”深度融合，积极探索云制造[11]模式，建设“云制造”服务平台，推动百家规模企业实施生产设备、流程、能力的数字化改造，做大做强数字产业。支持传统产业开展科技创新和商业模式创新。加大品牌、标准、专利战略实施力度，推动佛山制造向佛山创造、智造转变。

　　大力发展战略性新兴产业。继续实施产业链招商行动计划，瞄准重点区域、重点领域、重点项目开展有针对性的招商，着力引进光电、新材料、生物医药、新能源汽车等一批新兴产业龙头企业、重大项目和研发机构，形成战略性新兴产业集群竞争优势和技术领先优势。加快一汽大众佛山工厂、国星半导体、陆地方舟新能源汽车等一批重大项目建设。全面加强与国药集团的战略合作，推动佛山中医药产业转型升级，打造中国中药产业基地。

　　加快发展现代服务业。重点发展金融业、现代物流、工业设计、信息服务、商务服务等生产性服务业，推动现代服务业加速提效、集聚发展。积极申报省级服务外包示范城市。抓住“营改增”机遇促进现代服务业快速发展。围绕陶瓷、家具、家电、铝型材等优势支柱产业，加快培育建设一批商品国际采购中心。结合城市升级大力发展生活性服务业，加紧一批城市综合体和城市商圈建设，推动现代商贸业集聚发展。

　　优化发展现代农业。以科技创新推动现代农业发展，加大农业投入，增强农业综合生产能力和可持续发展能力。继续推进农业园区扩面提质工程，重点扶持发展“智慧菜篮子”基地，抓好农产品流通体系建设，保障农产品有效供给和质量安全。加强农业招商引资，培育发展农业龙头企业、新型农民合作组织和多元服务主体。实行最严格的耕地保护制度，妥善解决农村集体留用地遗留问题。

　　发展壮大民营企业。积极营造公平公正的民营经济发展环境，拓宽民营企业发展空间。制定和实施民营企业培育成长行动计划，加大引导扶持力度，培育一批具有国际竞争力、产值超百亿、超千亿的大公司、大企业集团和产业集群。鼓励有条件的民营企业通过兼并、收购、联合等方式做大做强。继续组织民企参加“北上西拓”、“广货网上行”等活动，引导民企积极开拓内需市场。推进服务企业活动常态化，帮助企业解决发展难题。扶持、引导企业加大技改投入，开展自主创新。建立健全企业家培育机制，打造一支庞大的、高素质的企业家队伍。

　　全面提高开放型经济水平。加大企业培育力度，逐步形成以技术、品牌、质量、服务为核心的出口竞争新优势，促进加工贸易转型升级。加快“走出去”步伐，积极组织企业参加各类国际展会,开拓国际新兴市场。推广国际商标有效注册，积极稳妥应对各类国际贸易摩擦。加强口岸和“大通关”建设，推动口岸改革和查验模式便利化。

　　以金融创新促进产业转型升级。实行“九个一”工程：一是建设一个广东省金融科技产业融合创新试验区，打造“一个核心区、若干辐射区”的融合发展平台。二是办好一个会议，把“金洽会”打造成为金融资本与科技、产业对接的高端平台。三是打造民间金融一条街，引进设立一批小额贷款、融资租赁公司等新型金融机构，推动PE和VC集聚发展。四是做好一个市场，积极申请区域性场外交易市场落户佛山。五是建好一张网，建设金融、科技、产业对接的网上金融超市，为企业提供全方位金融服务。六是吸引一批商业银行、担保公司、风险投资、私募股权、基金信托等创新型金融机构进驻佛山。七是搭建一个融资平台，包括区域集优债、中小企业私募债等融资创新平台。八是完善一套制度，包括企业上市“绿色通道证”制度、小额贷款、融资性担保公司评级制度等，构建具有佛山特色的金融体系。九是引进一批高端科技、金融人才。

　　打造优质载体集聚高端产业。制定佛山高新区产业发展规划及相关扶持政策，五区联动推进高新区实现跨越式发展，力争5年内进入全国国家高新区20强。加强广东金融高新区建设，推动走“金融后援基地+产业金融中心”新路子，力争打造成为国家级产业金融试验区。全力推进中德工业服务区建设，力争总体规划获国家、省批复通过。加紧申请制定《中德工业服务区条例》，建立与德国工业服务对接的政策制度体系，完善德国人才进驻的配套服务。狠抓重大项目实施，着力吸引优质项目进驻，打造中德两国高层次项目合作集聚区。加强中国南方生物医药城、广东工业设计城、高明新材料产业基地、欧洲工业园、新能源汽车产业基地、新媒体产业园、中国西南水都饮料（食品）基地等建设，为先进制造业和战略性新兴产业提供优质载体。

　　（三）着力推动城市内强外优，营造高品质城市生活空间

　　城市是区域经济发展的增长极。我们要大力实施“强中心”战略，推动区域融合互动发展，把城市打造成为高端产业和高端人才集聚的优质载体。

　　加强“一老三新”建设。加快魁奇路东西延线、季华路快速化改造、汾江路南延线、桂澜路南延线、海五路连结汾江路等路网建设，促进“一老三新”畅顺连接和一体化发展。加大中心城区规划及其管理市级统筹力度，促进中心城区一体化发展。佛山新城要加快核心区开发，着力推进苏宁广场、亚洲龙舟联合会总部、欧浦总部基地、企业家大厦、公共文化中心等项目建设。禅西新区要加强基础设施投入和公共资源配置，加快产业服务中心、专业人才生活园区、佛山市外国语（国际）学校等项目建设，推动新兴产业、社会资源和创新人才向西部集聚。桂城千灯湖片区要向更高品质发展，加快金融高新区Ｃ区、千灯湖公园等项目改造建设，树立城市东部门户新形象。

　　深化组团城市发展。加强全市规划统筹和土地储备，促进五区协调联动发展。推动区域之间资源整合和各领域合作。支持禅城“一老一新”核心区建设，提升中心城区城市品质和岭南文化独特魅力；支持南海东、中、西三大片区建设，形成同城异质、错位发展新格局；支持顺德新城高标准建设德胜河“一河两岸”、德胜总部商务区等重点工程，打造“阳光智城、岭南水乡、宜居家园”；支持高明西江新城“一心三轴线”建设，打造独具韵味的生态特色城区；支持三水新城加快建设，打造新城市中心、新产业服务中心、新生态宜居中心。

　　以“三旧改造”促进中心城区扩容提质。加大“三旧”改造实施力度，结合“三旧”改造推动历史街区建设和中心城区优化提升。打造城市北部门户，启动东货场功能改造提升，建设广佛商贸会展新中心，与桂城千灯湖片区联动发展。加强老城区保护和开发，推进祖庙-东华里、莲花路-升平路-筷子路、仁寿寺、梁园历史街区资源整合，重点对绿地、水系等生态环境进行恢复和整治。将市政府东侧和北侧地块打造成文化性地标节点，营造岭南文化集中展示空间。加快深村-两园（文华公园、亚艺公园）区段改造提升，促进季华路商圈提升能级和增强特色。加强佛山公园北园区段改造，启动东平路连接线工程，科学布局岭南大道开敞空间，开展地标项目规划设计，提升空间识别度。加快佛山新城区段开发建设，通过利益平衡引导周边村落积极参与改造，为新城建设和发展腾出空间。

　　打好城市升级攻坚战。推动城市升级由点到面、扩面提质，形成整体效应。加快广佛、广三、广珠西线等高速公路生态景观林带建设。抓好禅桂中心片区道路沿街建筑景观综合整治。继续推进城市道路“五位一体”改造，今年各区要建设1~2条改造示范路。加快农贸市场改造步伐，力争完成82个升级改造任务。各区要紧跟项目进度，狠抓项目落实。禅城区力争完成朝安路、卫国路等项目改造提升，推进佛山大道改造工程、佛山水道老城段滨河景观带、周尾围生态公园等项目建设。南海区力争完成怡海公园、听音湖片区水利及相关工程、南海博物馆、国道G321南海段改造等项目建设，推进平洲玉器街改造二期等项目建设。顺德区力争完成德胜河南岸滨水景观提升、德胜总部商务区景观改造、广东乐从钢铁世界现货区等项目建设，推进中国南方智谷B区、陈村花卉世界特色步行街区等项目建设。高明区力争完成秀丽河堤围景观综合整治、君御温德姆至尊酒店等项目建设，推进高明江滩滨河景观改造提升、中心城区四化工程等项目建设。三水区力争完成北江新区、北江体育休闲公园、城市休闲公园等项目建设，推进广海大道沿线景观改造提升等项目建设。

　　加强交通能源基础设施建设。把轨道交通建设作为城市可持续发展的重点，加快广佛轨道二期、广佛肇城际轨道及南海区新型轨道交通建设，积极探索TOD开发模式，开展轨道交通2、3号线TOD研究。抓紧推进佛山西站，广佛环线、佛肇城际轨道7个站点的“站场+社区”建设。加快高速公路项目建设，力争完成广明高速西延线建设任务，抓紧推进广明高速陈村至西樵段、肇花高速三水段、佛清从高速南段、江罗高速高明段等工程建设。加强能源、水利、通信等建设，推进气象现代化建设与考评，显著提升城市保障能力。重点补强佛山南部、北部电网，全面推进国家重点项目500千伏“西电东送”换顺线工程和东坡输变电工程建设。

　　着力提高城市管理水平。进一步加强城市管理考核工作，指导各区开展对镇（街道）的考核，推动城市管理考核实现全覆盖。对城市管理中的难点、黑点，泥头车撒漏、乱搭乱建、违规广告、占道经营等问题开展专项整治。禅城区重点规范夜间大排档管理，严厉打击非法营运，全面整治忠义路、普君市场等周边市容管理黑点。南海区重点加强桂城、罗村市容环卫景观整治提升，推进户外广告标准化建设。顺德区重点加强大良兴顺大道、德胜中路等路面养护及排水设施改造，对城市公园市政设施进行更新和绿化亮化提升。高明区重点对中心城区（A区）、高明大道沿线破损路面及公共设施进行修复，规范公路控制区范围内广告牌设置。三水区重点开展违章户外广告整治，提升中心城区公园、住宅小区保洁和绿化水平。佛山新城重点加强建筑工地周边环境和泥头车管理。拓宽数字城管覆盖范围，推动数字城管向镇（街道）延伸。加强公交站及换乘枢纽配建和公交港湾式停靠站建设，基本建成城市公交专用道网络。整合中心城区公交资源，优化提升中心城区交通环境。推进中心城区公交“六个一体化”[12]，有效提升公交管理和服务水平。

　　推动区域一体化发展。科学制定《实施<珠三角规划纲要>“九年大跨越”工作方案》。积极推动广佛同城化建设，重点加强金沙洲地区路桥对接、佛陈路东延线接番禺新桂路（海华大桥）、龙溪大道接海八路快速化改造、三善大桥扩建、魁奇路东延线衔接番禺区南大路等项目建设。做好与南沙开发的有效对接。推进广佛肇经济圈建设。继续加强佛港澳合作，提升合作质量和水平。

　　（四）着力推动生态文明建设，建设美丽佛山

　　建设生态文明，是关系人民福祉，实现佛山永续发展的长远大计，必须放在更加突出的位置，全力推进和落实。

　　探索低碳城市发展新模式。大力推广绿色建筑，建立建筑节能发展专项资金激励体系，研发与推广绿色建筑技术和材料。抓好禅城、顺德区低碳示范区建设，支持佛山新城开展绿色低碳城区建设技术集成与示范项目。大力推进节能降耗工作，通过推广应用先进节能技术、开展重点节能工程、发展循环经济、推行清洁生产，落实节能目标责任制、规范节能监管制度等措施，努力完成省下达的节能降耗目标任务。以全国电力需求侧管理[13]示范城市为契机，细化实施方案，深化天然气、可再生能源利用等分布式电源的规划发展。支持三水区建设国家新能源示范城市。

　　加强环境综合治理。加快使用传统能源小锅炉淘汰工作，鼓励使用天然气、液化石油气等清洁能源，逐步改善能源结构。强化电力行业脱硫脱硝设施建设，加强重点监管企业VOC防治，加快淘汰黄标车，铁腕整治黑烟车，逐步净化空气环境。落实汾江河等内河涌综合整治项目，加快污水处理厂及配套管网建设，实施最严格水资源管理及考核制度，推进饮用水源保护区标准化建设，持续改善水环境。开展畜禽养殖业污染治理，鼓励和支持规模化畜禽养殖场建设沼气池和废水处理系统。加强土壤重金属防治工作，强化管理危险物产生和经营单位，规范管理列入省严控废物管理污泥产生企业。深入开展环保专项执法行动，坚决打击各类环境违法行为。

　　提升生态绿化建设水平。积极创建国家生态市，全面启动国家环保模范城市复核工作。加强林业生态建设，继续推进生态景观林带、森林碳汇[14]、森林进城围城[15]工程建设，构筑生态安全体系。统筹整合全市绿道网资源，推动绿道文化建设。强化城乡园林绿化建设和管理工作，加大公园建设力度，逐步提高人均公园绿地面积。建设植物园、地质公园等专题园，提高城市品质。

　　统筹推动城乡生活垃圾处理。从源头分类、中转运输、终端减量“三管齐下”推进生活垃圾处理，加快建立和完善“村收集、镇中转、市区处理”的生活垃圾收集处理系统，力争形成垃圾分类处理的佛山模式。今年要全面完成生活垃圾压缩中转站的升级改造，实现“一镇一站”目标。制定餐厨垃圾处置实施方案，引进垃圾机械分选技术，推动终端处理设施改造提升，力争年底前禅城、南海、顺德区餐厨垃圾终端处理设施投入使用。加强宣传和政策引导，让市民养成生活垃圾分类处置的良好习惯。

　　（五）着力实施文化强市战略，增强城市文化软实力

　　文化是城市的魂和人民的精神家园。我们要大力推动社会主义文化大发展大繁荣，全面提高城市文化软实力。

　　提高全民道德和文化素质。深入开展社会主义核心价值体系宣传教育，弘扬中华优秀传统文化、岭南特色文化和新时期佛山人精神，营造积极向上的社会氛围。加强公民道德建设，积极开展全市文明礼仪标兵、道德模范、美德少年等评选活动，以及“微文明”等群众性精神文明创建活动，着力提升市民文明素质。

　　着力加强传统文化开发和保护。认真研究仁寿寺提升的定位、内涵和建筑形态，高标准、高质量做好寺院整体规划、配套建设、文物保护、管理提升工作，深入挖掘、整理佛山“佛文化”资源，致力打造佛教文化高地。全面启动祖庙古建筑群复建工程。支持祖庙、仁寿寺、岭南天地、梁园、南风古灶携手创建国家5A级景区。积极推动专题和行业博物馆建设。大力推进历史文化名镇、名村建设，坚持在改造中最大程度保护文物古迹。

　　进一步丰富人民精神文化生活。深入实施文化惠民工程，推动文化进园区、进社区，重点实施“魅力佛山·四季情韵”艺术惠民工程，办好佛山秋色欢乐节、龙舟文化节、佛山美食节、南风讲坛、“书香佛山”全民阅读系列活动等文化品牌。完善公益性文化、科普场馆面向社会免费开放机制，积极推行政府购买公共文化服务，合力打造市民喜闻乐见的“文化大观园”。

　　大力提高文化综合实力。深入实施文化产业发展规划，促进文化产业与城市升级、体育健身、旅游休闲相融合，打造特色文体旅游品牌。加大对工艺美术、创意设计、传媒影视、动漫游戏等重点产业扶持力度，依托特色文化产业园区，打造一批文化龙头企业和知名品牌。完善非物质文化遗产保护措施，加强传承人保护和培养，推动非遗项目走产业化发展道路。引导和鼓励社会资本以多种形式参与重大文化产业项目和文化产业园区建设。

　　深入推进“全国文明城市”创建。今年是创建全国文明城市的攻坚年，要针对未达标的重点指标、易反弹的重点工作、仍未解决的突出问题，确保有效投入，强化考评督办，推动常态管理，合力整改克难，力争年底全面达标，年后国检取得优异成绩。

　　（六）着力保障和改善民生，建设幸福佛山

　　坚持人民主体地位，以实现共同富裕、共享改革发展成果为导向,用心为民谋利，真心为民解忧，促进市民幸福感持续增强。

　　促进社会事业大发展。加强就业技能培训，深入推进创业带动就业，突出做好高校毕业生、城镇就业困难人员的就业工作。加强养老保险扩面征缴，完善居民医保市级统筹制度，建立职工医保与城乡居民医保制度衔接转换机制。继续稳步开展市、区两级教育综合改革试点，抓好学前教育三年计划攻坚，推进各级各类教育优质均衡发展，今年起在全省率先开展“中高职衔接”和“高职本科一体化”现代职教体系建设试点。认真实施医疗卫生服务体系建设三年提升计划，提高全市医疗卫生服务能力和水平。稳步推进公立医院改革，引导扶持民营医疗机构加快发展。启动佛山新城妇女儿童医院建设。广泛开展全民健身运动，加快公共体育设施建设。全面实施流动人口居住证“一证通”制度，完善积分入户政策。健全社会救助体系和社会福利制度，大力发展志愿服务事业，充分发挥红十字会在人道领域的助手作用。继续做好对口援建和扶贫开发“双到”工作。

　　扎实办好一批民生实事。加大保障性住房建设力度，今年新开工建设4350套，基本建成5883套。实施学前教育三年补贴制度及学前生均公用经费拨款制度，实施九年免费义务教育全覆盖。开展健康村（居）创建工作。推行城乡居民大病保险，逐步缓解因病致贫、因病返贫问题。建立我市未就业残疾人社会保险费用补贴制度。实施独生子女困难家庭特别扶助，解决独生子女伤残死亡家庭生活困难。对低保、五保、优抚、领取失业保险金人员实行联动机制补贴，减轻物价上涨对困难群众的影响。继续落实社区居家养老服务，推进家庭服务中心建设。全面落实职工生育保险办法，开展流动人口免费孕前优生健康检查。免除户籍人口基本殡葬服务费用，减轻群众殡葬负担。

　　全力建设平安和谐佛山。深入开展平安创建活动，加快智能警务建设，建立“三打”长效机制，加大对各类违法犯罪的打击力度，加强流动人口管理服务，大力推进社区民警进村居“两委”，确保维稳大局。深化普法工作，推进法治佛山建设。全面推进“国际安全社区”、“全国安全社区”试点创建和企业安全标准化建设，努力遏制重特大事故发生。严格农产品、食品、药品质量监管，推动安全食用农产品博览会升格为省部联办。健全信访工作机制，妥善化解社会矛盾纠纷。推进和谐劳动关系创建活动，逐步建立规范有序、公正合理、互利共赢、和谐稳定的劳动关系。

　　力争“两建”全省领先。按照统筹规划、重点突出、分步实施原则，全面推进社会信用体系和市场监管体系建设。着力加强对重点领域、重点人群和社会组织信用信息的记录、整合和应用，建设覆盖全市的公共联合征信系统，培育发展信用服务市场，逐步建立守信激励和失信惩戒机制。完善市场监管制度措施，推进市场监管综合执法体系改革，建立从源头到餐桌全链条、全过程、全覆盖的食品安全监管体系，在全省率先创建食品安全示范市。

　　（七）着力深化改革创新，增强发展活力与动力

　　改革创新是佛山永葆活力的关键。只有不断深化改革、锐意创新，佛山才能勇立发展潮头。

　　深化行政管理体制改革。深化完善大部门制改革，健全部门职责体系。科学理顺市、区事权关系，强化市级在重大规划、重大决策、重大项目布局等方面统筹力度。切实做好取消、转移、调整事项的衔接工作，加强对下放、转移职能事项的指导和监管。扎实推进中德工业服务区、佛山高新区、广东金融高新区三个综合改革试点。以“三个集中到位”[16]、“五个联合审批”[17]改革为核心，再造行政审批流程。全面推进行政审批标准化建设，实现无差别审批。全力推行网上办事服务，今年可网上审批事项申报率达到90%，网上办理业务量达到70%。

　　加强社会管理服务创新。加强行业协会商会、枢纽型社会组织、异地务工人员互助组织以及社会服务类、公益慈善类机构等社会组织建设，做好社会组织孵化培育、等级评估和向社会组织授权工作，确保政府职能“转得出、接得住、管得好”。加强城乡社区建设，努力将“小社区”打造成为加强社会建设、创新社会服务管理的“大舞台”。培育壮大专业化社工队伍。

　　深化经济领域改革。强化国资监管，整合国有资产，着力优化债务，提高国有企业的创利能力。加强市、区国资联动，推动市属国有企业土地盘活利用。深化财政综合管理改革，强化财政支出绩效管理，着力构建持续稳健、保障有力的财政体系。

　　深化农村综合改革。稳步推进村居“政经分离”、“政社分离”[18]改革，强化村居自治功能。支持南海区探索推动农村集体经济组织市场化、公司化改造。加强农村集体“三资”管理，夯实“两个平台”基础,有效保障和发展村民利益。支持顺德区理顺基层组织关系，构建自治有序、长治久安的基层治理格局。积极探索农村公共产品多元化供给机制，按照“谁投资、谁受益”原则，引进社会资源兴办农村公益事业，推动农村公共服务全覆盖。

　　（八）着力提升理政水平，努力建设人民满意政府

　　面对人民群众的新期盼和历史赋予的新使命，我们要不断加强政府自身建设，切实提高理政能力和水平。

　　提高依法行政能力。开展依法行政工作考评，加快法治政府建设。规范行政执法行为，健全与大部门制相适应的行政执法运行机制。认真做好行政应诉工作，今年全市行政机关负责人出庭应诉率力争达到95%。完善行政复议委员会制度，进一步提升化解行政争议的能力。加强政府信息公开，通过政府网、政务微博等途径，及时准确向社会公开政府信息。

　　加强科学民主决策。自觉接受人大、政协、公众和舆论监督，坚持专家咨询、社会听证、决策评估和集体决策机制，落实重大决策合法性审查制度。推行重大决策跟踪反馈和责任追究，确保民主决策的科学性和准确性。

　　提升政府行政执行力。加强市区镇（街道）三级管理有效衔接，促进各项政策落实到位。实施重大产业项目落实推进联席会议制度，切实帮助项目解决各种难题，促进项目早日落户、开工和投产。完善督查督办机制，加大考评和问责力度，推动重点工作项目化、检查监督常态化。切实加强作风建设，坚持说实话、办实事、求实效。全面实施政府绩效管理，提升工作效能。

　　加强廉洁佛山建设。加强重点领域、重点环节和重点岗位廉政风险防控，以水利工程为试点，继续推进公共资源交易平台和监管体系建设。探索建立商业贿赂“黑名单”制度，推进社会领域防治腐败工作，构建反腐新格局。加强党员干部廉洁文化教育，增强崇廉尚洁意识。大力推进网上办事和电子监察，严肃查处损害群众利益的违纪违法案件，让权力在法定轨道运行。

　　各位代表！佛山正处在转型升级和创新发展的关键时期，面对新的目标任务，我们深感责任重大，使命光荣。让我们在党的十八大精神指引下，在省委、省政府和市委的正确领导下，紧紧依靠全市人民，创新改革，砥砺奋进，为加快转型升级、建设幸福佛山而努力奋斗！
